# Supplementary material for: Yeast One-Hybrid Screening for Transcription Factors of IbbHLH2 in Purple-Fleshed Sweet Potato
Source: Genes (Basel). 2023 May 5;14(5):1042. doi: 10.3390/genes14051042 (PMC10218299; doi:10.3390/genes14051042)
Supplement: Supplementary file 1 [file genes-14-01042-s001.zip › Supplemental sequences.pdf]

pIbbHLH2

CGGCCGCTTTATTGGTGAAGGGTAAGACAAGAATTTGTAGCCACGCATAA
CTTATAATCTTAAGTATGATGATCATATTTTATTCATTAAGCTACTCTTTTT
GGAGTAACCATATCATGTTTGTCTAATTTTTCATTTAATAGCTAGAGAGAA
TATTTAAAGATCATAAATTTGATTTTTGTCAAAATTTTCTTAGTCGATAGCT
GCACAAAATTTTCTTAGTGTAGTTTACCTCTGCAGTGTGGTCTGCAAGTTG
TTACATATTAATAAAATTTATTCAAATAAACTCTCAGATAATAAAATGGG
GTTTCTTATAAAAAAAATAAAAATTAAAAATAAACTATAGGAACAGAAAA
TAGGCATACTCTTGATAAATAAGAGATGATGAAGAATTGAAGACCTCTTG
TGCGCAGAGTGCACAAGTAAGCGATGAAAACTTGAAGACAGATAAGGCA
ACTTACAATTTACCTCTACTAGAAATTCTTAGGTAGTATGTGGTTCTGAAC
GTTTAGAATTAATTAGAGTTGTCGCATGGTAGCTTGATCAATTGGTCACAT
GTGTGAAGTTTGGAAGAAGAAATCAAGGATCGAGTCTCATCAGTGGCAAT
GTAGGAGCAACCCCTTAAAGTGAGGGGGTCCTTGTGCCTGGTTTAGTCCA
CTGAGGCTCAAATCCACCCCCATCCACCCCATATGAAGGTGAAACCGGGT
GTCACTAAATCACAAGTCTTTGACAGAAGAATTAAATAGAGCTGAAAAAC
CTAACTAAATTTATAATAAACAAAAAAAAAAAAGTGACACTAGTATAGTA
ATATGATACACGTGGGACACATAAAAGGCAAGGACAAAAACCTAATCTT
GAATTCTCCTATTTTGTCGTCTCTTTCCCAGTCCCAATACCCGACCGGTTG
ACACCAACCAGTCAAAATCCAACTCCCCGACAAACAAATAAATTCAACCT
TAACCCCTCTCCGGTCTGCAACTCTTAATTTCATATGTAAACCACTCTGTC
TCACATCTTTCCC

IbERF1

ATGAATTCAGGATTCTCCTCCGATTCATCGCCGGAATATTTCCCGTGGGAT
TGCCGGAATTTCGACAGCTCCCTCCCGTTCAACGTTGATGACTCCGAAGA
AATGCTCCTCTTCGGGGTTCTGTCTCAGGCGGCGGCGCGAGAGAACTCCG
AGACCAATTCCTCGGATTACAGCGTGAAGGAAGAGGAGGTGACCTCGGA
GACTCCGAAGAAGGAGCAGCGGTCGTTCCGCGGCGTGAGGCGGCGGCCC
TGGGGGAAATTCGCGGCGGAGATAAGGGATTCGACGAGGAACGGGATAA
GGGTTTGGCTGGGAACCTTCGACAAAGCAGAGGAGGCGGCTCTGGCCTAC
GACCAAGCGGCGTTCTCGATGCGGGGGCCCATGGCGGTCTTGAACTTCCC
CGTGGAGACTGTCCGGGAATCTCTCCGGGAAATGAGGTGCCCCGTCGAGG
AGGGCGGCTCTCCCGTGGTGGCGCTCAAGCGGAGACACTCCTTGCGGCAG
CGGGGATTGAACCGGAGGAGCGGCAAACGGCGGGAGGTGAAGGTGGAGA
ATGTGGTGGTGTTTGAGGACTTGGGTGTTGATTACTTGGAACAACTTTTGA
GTTCAAGTGGGACTACAACAACATCATCATCATCATCAATTACTCCTAATC
CCTTGGTTTAA

IbERF10

ATGGCTCCCAAGGAGAAGGGGACGGCGGTGGCGGTTGCGGCGGCGAAAG
TACATGAGATTCGGAAGGAGCAGGTGCATTTTAGGGGCGTGAGGAAGAG
GCCGTGGGGGAGATATGCGGCGGAGATTAGGGATCCCGGGAAGAAGAGC
CGCGTTTGGCTCGGGACGTTTGATACGGCGGAGGAGGCGGCTAGGGCGTA
CGATAAGGCGGCGAGGGAGTTTCGGGGTTCCAAGGCGAAGACTAATTTTC
CGGTGCCGGAGGATCTCCTTCCGCTCAATTTCGCCGCGCAATTGCACGTGA
AGAATAGCGTGTGTGAGAAGAACGCCGGCGGGAGTCCGAGCCCGAGCAG
TACTGTTGAGTCGTCGAGCGGCGGCGGCGGCGGAGGCTGTGGTCGTATCT
CGCCGGCGGTGATGGCGGACTCGTCGCCTCTGGATCTAAGCCTAGGCGGC
CGTGCGCCGGTGATGTTTCCGATACAGAATCACCCCCAGCGCGTGGTTCA
TCCCATGGTTCCCGGCGCGTACCCGGCCGGGGTCTATCCCGTTAGCCAGAT
TGTTTATCTCAACGCGCTCGCCCGTGCCGTGGCGGTGAACCCTCCGCTCAA
CCGGCAAACCGAGACGCTCAATTTCTTCGCCCCCAACGCCAACTGTGGGG
ACCACAGCGACTCCGACTCTTCATCGGTCATTGACTTGAACGCCGGCTATG
TCGAACCGCGAAAGGGTGGGCCCATTAACAATTTCGATCTCAACCACCCA
CCGCCAGAGAGTCTCGGAACCGGCGCTCTCTAA
