# Supplementary material for: Yeast One-Hybrid Screening for Transcription Factors of IbbHLH2 in Purple-Fleshed Sweet Potato
Source: Genes (Basel). 2023 May 5;14(5):1042. doi: 10.3390/genes14051042 (PMC10218299; doi:10.3390/genes14051042)
Supplement: Supplementary file 1 [file genes-14-01042-s001.zip › Supplemental Tables.pdf]

Supplemental Table S1 The primer sets used in this study

Name	Sequence(5'-3')
	**Yeast one-hybrid screening**
pbHLH2-1F	AAATGATGAATTGAAAAGCTTGCATAACTTATAATCTTAAGTATGATGATCATAT
pbHLH2-1R	GTCGACAGATCCCCGGGTACCCTACCTAAGAATTTCTAGTAGAGGTAAATTGTA
pbHLH2-2F	AAATGATGAATTGAAAAGCTTTATGTGGTTCTGAACGTTTAGAATTAAT
pbHLH2-2R	GTCGACAGATCCCCGGGTACCGGGAAAGATGTGAGACAGAGTGG
pAbAi-F	CAAGTTTGTTTGCTTTTCGTGC
pAbAi-R	GCTGGAATGATGAATAAAGTGCC
	**Yeast one-hybrid assay**
ERF1-ADF	GCCATGGAGGCCAGTGAATTCATGAATTCAGGATTCTCCTCCGA
ERF1-ADR	CAGCTCGAGCTCGATGGATCCTTAAACCAAGGGATTAGGAGTAATTG
ERF10-ADF	GCCATGGAGGCCAGTGAATTCATGGCTCCCAAGGAGAAGGG
ERF10-ADR	CAGCTCGAGCTCGATGGATCCTTAGAGAGCGCCGGTTCCG
pGADT7-F	CGATGATGAAGATACCCCACCAAACC
pGADT7-R	ACTTGCGGGGTTTTTCAGTATCTACG
	**Yeast two-hybrid (Y2H) assay**
ERF1-BDF	ATGGCCATGGAGGCCGAATTCATGAATTCAGGATTCTCCTCCGA
ERF1-BDR	CCGCTGCAGGTCGACGGATCCTTAAACCAAGGGATTAGGAGTAATTG
ERF10-BDF	ATGGCCATGGAGGCCGAATTCATGGCTCCCAAGGAGAAGGG
ERF10-BDR	CCGCTGCAGGTCGACGGATCCTTAGAGAGCGCCGGTTCCG
pGBKT7-F	TAATACGACTCACTATAGGGCGA
pGBKT7-R	GACTCTTAGGTTTTAAAACGAAAA
	**Dual-luciferase assay**
pb2-1-0800F	CTATAGGGCGAATTGGGTACCCGGCCGCTTTATTGGTGA
pb2-1-0800R	TGTTTTTGGCGTCTTCCATGGCTACCTAAGAATTTCTAGTAGAGGTAAATTGTA
p0800-LUC-F	CAAGGCGATTAAGTTGGGTAAC
p0800-LUC-R	CTCTCCAGCGGTTCCATCTTCC
ERF1-62-SKF	AGGACAGCCCAAGCTGAGCTCATGAATTCAGGATTCTCCTCCGA
ERF1-62-SKR	GGTACCGGGCCCCCCCTCGAGTTAAACCAAGGGATTAGGAGTAATTG

ERF10-62-SKF	AGGACAGCCCAAGCTGAGCTCATGGCTCCCAAGGAGAAGGG
ERF10-62-SKR	GGTACCGGGCCCCCCTCGAGTTAGAGAGCGCCGGTTCCG
p62-SK-F	CATATAAGGAAGTCATTTCATT
p62-SK-R	TCCCTTATCGGGAAACTACTCA
**Subcellular localization analysis**	
ERF1-1300F	TTTTCTGATTAACAGGGATCCATGAATTCAGGATTCTCCTCCGA
ERF1-1300R	GCTCTCGAGACTAGTGGTACCAACCAAGGGATTAGGAGTAATTGATG
ERF10-1300F	TTTTCTGATTAACAGGGATCCATGGCTCCCAAGGAGAAGGG
ERF10-1300R	GCTCTCGAGACTAGTGGTACCGAGAGCGCCGGTTCCGAG
pCambia1300-F	TACTCTTCGATTTGTGATTTC
pCambia1300-R	CATCGCAAGACCGGCAACAGG
**Real-time quantitative PCR**	
Q-G14-F	GTGCGGAAACTGCGACTGC
Q-G14-R	CGTCCATCTTGCTTCCCTTCCT
Q-MYB1-F	ATGGTTATTTCATCTGTATGGTCGG
Q-MYB1-R	CTCAAAAGTTGGTCTTCTTCTTCG
Q-bHLH2-F	TGAACTGGGAACAACGGAAAGGG
Q-bHLH2-R	ATGATGATGGTGGTGGGGATGGGG
Q-WD40-1F	GAGAAAGGCGTGGTGGAAACCCA
Q-WD40-1R	AACCGACCCGTCAGCCGACACTGAA
Q-ERF1-F	AACGGGATAAGGGTTTGGCTGGG
Q-ERF1-R	CGGACAGTCTCCACGGGGAAGTT
Q-ERF10-F	GAGGGAGTTTCGGGGTTCCAAGG
Q-ERF10-R	GGCGTTCTTCTCACACACGCTATTCT
Q-CHS-F	AAAACCTGAAAAACTCCGAGCCACG
Q-CHS-R	CAAACCCAAACAGCACACCCCAC
Q-CHI-F	GAAGGTGTCGGAAAACTGCGTTG
Q-CHI-R	GGGGTGATTGAGTGAAGAAAATAGAGG
Q-F3H-F	TTCATCGTTTCCAGCCATCTCCA

Q-F3H-R	TTTTCCGTTACTGCCCTCCACCC
Q-F3’H-F	CTGTGGAGTGGGCATTCGCAGAA
Q-F3’H-R	AGAGAGTGGGGTGGATGGGTGTAGC
Q-ANS-F	AATAATGCTAGTGGGCAGCTTGAGTG
Q-ANS-R	AAGGAGTTTTAGGCCAAATGGAGAGA
Q-DFR-F	CTGCTGGCTTTATCGGCTCCTGGT
Q-DFR-R	TGTCAAATTCGTGTCCGCTTTCG
Q-UF3GT-F	GCGTTCCTTGGATTTCTTTTTGG
Q-UF3GT-R	TGACATTCCTGGGATTACTTTCAGCT

Supplemental Table S2 Information of genes interacted with *pIbHLH2-1* by yeast one hybrid screening

NO	Gene	Accession	Length(bp)	NO	Gene	Accession	Length(bp)
1	*UR5GT*	AT4G09810.1	2176	112	*hsp70*	AT3G09440.1	3593
2	*LLG3*	AT2G22821.1	829	113	*L30*	AT2G44120.2	1738
3	*PCS*	AT3G54020.1	2639	114	*SAP*	AT5G39090.1	1680
4	*M17*	AT2G41260.2	1247	115	*SAP2*	AT5G66840.1	2683
5	*MBOAT*	AT1G57600.1	4711	116	*RBP47B*	AT1G28330.5	1655
6	*RVE6*	AT5G52660.2	3273	117	*PP2C*	AT1G47380.1	3721
7	*PGP10*	AT1G49320.1	1784	118	*GR*	AT5G46730.1	1246
8	*CHC1*	AT3G11130.1	9917	119	*NGA4*	AT4G01500.1	1730
9	*NAC*	AT4G35520.1	6040	120	*RRT4*	AT1G14020.1	2656
10	*ARM*	AT4G16490.1	2547	121	*EBF2*	AT5G47040.1	5915
11	*RXF26*	AT1G06980.1	1039	122	*LTP12*	AT3G51590.1	1057
12	*SNF7*	AT2G19830.1	1352	123	*SRC2*	AT1G09070.1	1606
13	*DPA*	AT5G02470.1	2618	124	*ADF6*	AT2G31200.1	1458
14	*AH*	AT3G23600.1	2398	125	*ZincRP*	AT3G16080.1	1181
15	*MPK17*	AT4G25900.1	2400	126	*ACA9*	AT3G21180.1	6890
16	*AGD12*	AT4G21160.1	2692	127	*GH9C2*	AT1G64390.1	4213
17	*MC4*	AT1G79340.1	2052	128	*LTI6B*	AT3G05890.1	1368
18	*DJA5*	AT4G39960.1	2885	129	*HINT4*	AT4G16566.1	2349
19	*MYB3R4*	AT5G11510.1	5137	130	*RCE1*	AT4G36800.1	2104
20	*G6PD6*	AT4G09520.1	2320	131	*PS*	AT4G39280.1	3514
21	*SLK2*	AT5G62090.1	4207	132	*RING*	AT1G13195.1	2096
22	*TIN1*	AT5G64510.1	1982	133	*RF*	AT3G10915.5	1814
23	*GF*	AT5G57500.1	1543	134	*HDG2*	AT1G04353.1	1945
24	*UBQ2*	AT2G36170.1	1280	135	*PBL38*	AT2G39110.1	2239
25	*RER1C1*	AT2G23310.1	1937	136	*PFK7*	AT5G56630.1	3160
26	*AAH*	AT4G20070.1	3384	137	*HER2*	AT5G63620.1	2423

27	*UNE12*	AT4G02590.1	3054	138	*FQR1*	AT4G36750.1
28	*PDC3*	AT5G01330.1	2657	139	*AMT2*	AT2G38290.1
29	*rRNA*	AT3G41768.1	1808	140	*BBE23*	AT1G51810.1
30	*MEM1*	AT1G48950.1	3527	141	*NH*	AT5G12040.1
31	*DCD*	AT3G27090.1	2316	142	*AUXIL*	AT1G30280.1
32	*GBF1*	AT4G36730.1	2877	143	*PSBK*	ATCG00070.1
33	*L18e/L15*	AT1G70600.1	959	144	*TSD2*	AT1G78240.1
34	*A20/AN1*	AT1G51200.1	2354	145	*ATG3*	AT5G61500.1
35	*MAC5A*	AT1G07360.1	2607	146	*ASP3*	AT5G11520.1
36	*SRN19*	AT5G61820.1	2634	147	*AUG1*	AT2G41350.2
37	*tRNA*	AT4G16510.1	3237	148	*PP2A-3*	AT2G42500.1
38	*RRM*	AT1G22910.3	2576	149	*SPPL1*	AT4G33410.1
39	*SOQ1*	AT1G56500.1	7598	150	*PES*	AT5G14520.1
40	*Gh35*	AT2G04060.1	3028	151	*bHLH093L*	AT5G65640.1
41	*WSD6*	AT3G49210.1	3481	152	*AIG1*	AT1G68810.1
42	*UGT73B3*	AT4G34131.1	1762	153	*BRXL4*	AT5G20540.1
43	*RHO*	AT5G22400.1	2905	154	*ER*	AT5G10780.2
44	*TCP-1*	AT1G24510.1	2976	155	*NCBP*	AT5G18110.1
45	*RRM*	AT4G35785.5	2380	156	*GATA2*	AT2G45050.1
46	*CBF5*	AT3G57150.1	2261	157	*YUC2*	AT3G48500.2
47	*DIN9*	AT1G67070.1	2815	158	*SCAMP2*	AT1G11180.2
48	*Hsp 70*	AT3G09440.1	3593	159	*APUM24*	AT4G00891.1
49	*ABF4*	AT3G19290.3	3082	160	*RP14P*	AT1G04480.1
50	*BAG*	AT1G62305.1	2802	161	*RPS5*	AT5G18380.1
51	*EMB2769*	AT3G13200.1	2074	162	*ATG18G*	AT1G03380.1
52	*RBP47B*	AT3G19130.1	2926	163	*TP*	AT3G01130.1
53	*nst1*	AT1G80700.1	1559	164	*DUF726*	AT4G36210.3
54	*KHE*	AT1G53760.1	2207	165	*RXF26*	AT1G58430.1
55	*RBL2*	AT1G63120.1	2336	166	*MSD1*	AT3G10920.1

56	*HP*	AT1G22065.1	770	167	*EB*	AT3G04480.1
57	*CSLC4*	AT3G28180.1	3555	168	*EIF2*	AT2G39990.1
58	*EMB2016*	AT3G05680.2	12086	169	*BHLH129*	AT2G42280.1
59	*FDA10*	AT2G16365.1	5158	170	*IRP1*	AT3G07170.1
60	*DR*	AT4G24380.1	1342	171	*SAP1*	AT1G03230.1
61	*ACA9*	AT3G21180.1	6890	172	*LPPGAMMA*	AT5G03080.1
62	*SINAT3*	AT3G61790.1	2137	173	*SPPL5*	AT1G05820.1
63	*PK*	AT5G61570.1	2601	174	*KIN3*	AT2G17220.1
**64**	***ERF10***	**AT5G44210.1**	**1145**	175	*CYCD4*	AT5G65420.3
65	*CCHC*	AT3G42860.1	2086	176	*PDS4*	AT2G07717.1
66	*EXPR*	AT4G17030.1	1729	177	*AGD6*	AT3G53710.1
67	*ARC*	AT4G09360.1	3095	178	*PERK1*	AT3G24550.1
68	*PPR*	AT5G18475.1	1884	179	*RPS6*	AT4G31700.1
69	*RUB1*	AT4G36800.1	2104	180	*SINAT3*	AT3G61790.1
70	*RP40*	AT1G72370.1	2087	181	*CDS*	AT2G47010.1
71	*DRM1*	AT1G28330.5	1655	182	*TP-SH3*	AT1G09590.1
72	*SP*	AT1G69980.1	1858	183	*WD40*	AT5G14050.1
73	*MICU*	AT4G32060.1	2668	184	*DE*	AT4G19110.2
74	*PCAP1*	AT4G20260.4	2757	185	*GTF61*	AT3G18170.1
75	*RING*	AT1G13195.1	2096	186	*DUF760）*	AT5G48590.1
76	*MAET*	AT1G33110.1	3999	187	*TIM17-2*	AT2G37410.1
77	*PA*	AT4G30020.1	4821	188	*GOR*	AT1G30090.1
78	*VDAC3*	AT5G15090.1	2281	189	*SIB1*	AT3G56710.1
79	*PFK7*	AT5G56630.1	3160	190	*ARAC*	AT2G17800.1
80	*CDCP2*	AT4G36910.1	2854	191	*YLS8*	AT5G08290.1
81	*QR*	AT4G36750.1	2015	192	*CRA-31*	AT5G42570.1
82	*PGP19*	AT3G28860.1	7574	193	*LRP*	AT3G47090.1
83	*SS2*	AT3G01180.1	3867	194	*ACS12*	AT5G51680.1
84	*AS*	AT2G21390.1	4967	195	*PDC1*	AT4G33070.1

85	*MPK8*	AT1G18150.2	3447	196	*DUF788）*	AT2G23940.1
86	*AUXILIN*	AT1G30280.1	2048	197	*LPK*	AT4G37830.1
87	*COS*	AT4G37830.1	1311	198	*AH*	AT2G21390.1
88	*TSD2*	AT1G78240.1	3846	199	*SINAT3*	AT3G61790.1
89	*G1B*	AT3G53930.2	4493	200	*UPL4*	AT5G02880.1
90	*ASP3*	AT5G11520.1	2909	201	*ACLA-1*	AT1G10670.3
91	*GLTP1*	AT2G33470.1	2445	202	*AS2*	AT5G20060.2
92	*AMM*	AT5G64030.1	4608	203	*DCL*	AT1G45230.1
93	*DUF569*	AT1G69890.1	1861	204	*RBCL*	ATCG00490.1
94	*BEX1*	AT2G47170.1	2245	205	*RAB18*	RAB18
**95**	***ERF1***	**AT3G23240.1**	**988**	206	*UBC2*	AT2G02760.1
96	*GLDH*	AT3G47570.1	3535	207	*LTPG2*	AT3G43720.1
97	*BRXL4*	AT5G20540.1	4250	208	*RLP57*	AT5G65830.1
98	*MYB3R4*	AT5G11510.1	5137	209	*HP*	AT5G49260.1
99	*SAMDC*	AT3G02470.1	2445	210	*AP2S*	AT1G47830.1
100	*TIN1*	AT5G64510.1	1982	211	*AIR12*	AT3G19970.1
101	*PLIM2B*	AT1G01780.1	1485	212	*NDA2*	AT2G29990.1
102	*NFD3*	AT1G20696.2	2047	213	*AMDM*	AT5G64030.1
103	*DL*	AT1G75860.1	1823	214	*TLP5*	AT1G43640.1
104	*COX11*	AT1G02410.1	2104	215	*CAT2*	AT4G35090.1
105	*PGD2*	AT3G02360.2	2120	216	*UBQ4*	AT5G20620.1
106	*CM2*	AT5G10870.1	1972	217	*VTC2*	VTC2
107	*DCD*	AT3G27090.1	2316	218	*CCR1*	AT1G15950.1
108	*UGT73B3*	AT4G34131.1	1762	219	*ELI3*	AT4G37990.1
109	*TWN2*	AT1G14610.1	6381	220	*CSC1*	AT4G22120.1
110	*RRM*	AT4G35785.5	2380	221	*OPD*	AT1G62250.1
111	*AMDM2*	AT5G64030.1	4608	222	*RABG3D*	AT1G52280.1
